# Effect of the Number of Dark Days and Planting Density on the Health-Promoting Phytochemicals and Antioxidant Capacity of Mustard (*Brassica juncea*) Sprouts

**DOI:** 10.3390/plants11192515

**Published:** 2022-09-26

**Authors:** Zhiqing Li, Hongmei Di, Wenjuan Cheng, Guanru Ren, Yi Zhang, Jie Ma, Wei Ma, Jiao Yang, Huashan Lian, Xiaomei Li, Zhi Huang, Yi Tang, Yangxia Zheng, Huanxiu Li, Fen Zhang, Bo Sun

**Affiliations:** 1 College of Horticulture, Sichuan Agricultural University, Chengdu 611130, China; 2 Institute of Agricultural Resources and Environment, Tianjin Academy of Agricultural Sciences, Tianjin 300384, China; 3 College of Forestry, Sichuan Agricultural University, Chengdu 611130, China; 4 Bijie Institute of Agricultural Science, Bijie 551700, China; 5 School of Agriculture and Horticulture, Chengdu Agricultural College, Chengdu 611130, China; 6 Rice and Sorghum Research Institue, Sichuan Academy of Agricultural Sciences, Deyang 618000, China; 7 Vegetable Germplasm Innovation and Variety Improvement Key Laboratory of Sichuan, Chengdu 610300, China

**Keywords:** mustard (*Brassica juncea*), sprouts, dark, planting density, health-promoting phytochemicals, glucosinolate, antioxidant capacity

## Abstract

Mustard is an edible vegetable in the genus *Brassica* with tender and clean sprouts and short growth cycles that has become a rich source of nutrients required by humans. Here, the effects of dark exposure duration and planting density on the health-promoting phytochemicals and the antioxidant capacity of mustard sprouts were evaluated. The content of soluble sugar, soluble protein, chlorophyll, and carotenoids and the antioxidant capacity of mustard were higher in the two-day dark treatment; the content of indolic glucosinolates was also more affected in the dark day experiment than in the planting density experiment. The soluble sugar, soluble protein, and aliphatic and total glucosinolate levels were higher when sprouts were grown at high densities (6–7 g per tray); however, no significant variation was observed in the content of chlorophyll and carotenoids and the antioxidant capacity. The results of this study show that the optimum cultivation regime for maximizing the concentrations of nutrients of mustard plants is a planting density of 6 g of seeds per tray and a two-day dark treatment.

## 1. Introduction

Mustard (*Brassica juncea*) is a widely consumed, nutritious vegetable in the genus *Brassica* [1]. It is mostly cultivated for use as a fresh vegetable because of its high concentrations of bioactive components, such as chlorophyll, carotenoids, ascorbic acid, phenolic compounds, and glucosinolates, and many studies of these biochemical compounds in mustard have been conducted in recent years [2,3,4]. The consumption of sprouts is increasing, and this increase has been largely driven by a growing trend in healthy eating habits among consumers [5,6]. Sprouts have been shown in many studies to be more nutrient-dense than ungerminated seeds or even mature vegetables [5]. The levels of these health-promoting nutrients in sprouts are greatly affected by the growth environment and culture conditions [7,8]. For example, a previous study has shown that UV-B illumination treatment might increase the content of carotenoids and glucosinolates during the germination of white mustard sprouts [6]. Low temperature (8 °C) has been shown to significantly decrease the content of carotenoids and flavonoids of kale sprouts and increase the content of total glucosinolates [9]. However, very little research has examined the effects of dark treatment and planting density on the concentrations of health-promoting phytochemicals and the antioxidant capacity of sprouts.

There are two essential pathways in plant development: photomorphogenesis, which is triggered by light, and skotomorphogenesis, which is triggered by darkness [10]. Several previous studies have examined the effects of light factors on plants, especially light intensity, photoperiod, and light quality, because of their major consequences on plant growth and development [6,11,12]. However, the normal growth and development of plants can be disrupted if the darkness cues for inducing skotomorphogenesis are not appropriate [11,13]. The percentage of shading has a major effect on the germination and hormone content of Palmer amaranth seeds [14]. The accumulation of glucosinolates is enhanced in Pale Green and Purple Kohlrabi sprouts when dark conditions are appropriate [15]. Continuous irradiation, in which plants are exclusively exposed to light, causes the photosynthetic efficiency and nutrient quality of adult lettuce to decrease sharply [11]. These findings indicate the importance of exposure to darkness. This is especially the case for sprouts. Endogenous gibberellins (GAs) have been shown to play a key role in controlling the hypocotyl elongation of sprouts in skotomorphogenesis [16]. Given that the amount of GAs that accumulate in sprouts varies with the number of days that plants are exposed to darkness, studies are needed to determine the optimal number of days that sprouts should be exposed to darkness.

Planting density is also a key variable affecting the growth and development of plants [17]. Numerous studies have shown that cultivation at appropriate planting densities can enhance the yield of several crops, such as Brussels sprouts [17], spinach [18], and beetroot [19]. However, increases in planting density are not directly proportional to increases in nutritional quality, as the relationship between planting density and nutrients can be complex [18,20,21]. For example, low planting density increases the content of soluble sugar, protein, chlorophyll, carotenoids, total phenols, and flavonoids in cucumber [21]. The level of ascorbic acid and the antioxidant activity of red cabbage are highest under a medium planting density (125,000 plants ha^−1^) compared with low (100,000 plants ha^−1^) and high (166,700 plants ha^−1^) planting densities [20]. The lowest marketable quality of spinach, which is determined by the content of β-carotene, vitamin C, and nitrate, was observed when it was cultivated at the lowest planting density (800,000 seeds ha^−1^) [18]. In sprouts, a low seedling density can result in the sparse growth of sprouts and low yield. By contrast, high seedling density can result in uneven growth and decreases in biomass and nutritional quality due to limitations in matrix nutrients and space [17,22].

Given that the number of dark days and planting density both have substantial effects on the growth and quality of sprouts, studies are needed to identify the optimal cultivation conditions for the growth of sprouts. Here, the effects of the number of dark days and planting density on the health-promoting phytochemicals and antioxidant capacity of mustard sprouts were examined.

## 2. Results

### 2.1. Soluble Sugar and Soluble Protein

The soluble sugar content was highest in D2 (158.36 mg g^−1^); the soluble protein content was similar in D3 (109.68 mg g^−1^) and D2 (109.14 mg g^−1^) (Table 1). D2 was thus optimal for the production and accumulation of soluble sugar and protein.

Meanwhile, the soluble sugar and soluble protein content was highest in P4 (152.65 mg g^−1^) and P3 (113.35 mg g^−1^), respectively (Table 2), which indicated that high planting density enhanced the soluble sugar and protein content.

### 2.2. Chlorophyll and Carotenoids

The chlorophyll content was highest in D2 (7.29 mg g^−1^), and it was 5.72%, 13.17%, and 27.83% higher in D2 than in D1 (6.89 mg g^−1^), D3 (6.44 mg g^−1^), and D4 (5.70 mg g^−1^), respectively. The carotenoid content was highest in D1 (0.64 mg g^−1^), and there was no significant difference in the carotenoid content between D2 (0.63 mg g^−1^) and D1 (Table 1). D2 had a positive effect on the accumulation of photosynthetic pigments. However, there was no significant effect of planting density on the level of chlorophyll and carotenoids (Table 2).

### 2.3. Ascorbic Acid

The ascorbic acid content was highest in D4 (1.41 mg g^−1^), followed by D2 (1.31 mg g^−1^), and there was no significant difference in the ascorbic acid content between D4 and D2 (Table 3).

The ascorbic acid content was higher in P3 (2.52 mg g^−1^) and P4 (2.61 mg g^−1^) than in P1 (2.24 mg g^−1^) and P2 (2.07 mg g^−1^) (Table 4), which indicated that the ascorbic acid content was higher at high planting densities.

### 2.4. Proanthocyanidins, Flavonoids, and Total Phenolics

The content of proanthocyanidins (5.95 mg g^−1^), flavonoids (14.92 mg g^−1^), and total phenolics (16.05 mg g^−1^) was highest in D2 (Table 3), demonstrating that D2 was optimal for maximizing the content of the above three antioxidants.

The content of proanthocyanidins was highest in P3 (6.39 mg g^−1^), and no differences were observed in the content of flavonoids and total phenolics among the four planting density treatments (Table 4).

### 2.5. Antioxidant Activity

High levels of ABTS (47.97 mg g^−1^) and FRAP (0.15 mmol g^−1^) were observed in D4, and no significant differences were observed in ABTS and FRAP among the three remaining treatments (Table 3). Planting density barely made any difference to the antioxidant activity (Table 4).

### 2.6. Glucosinolates

**Figure 1:**
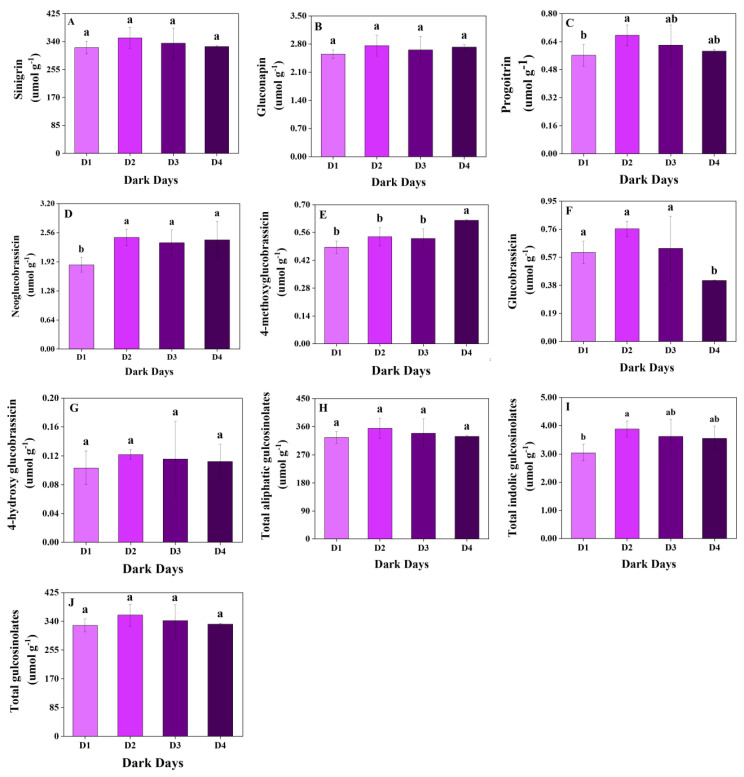
Glucosinolate content under different days of dark treatment in mustard sprouts. Data are expressed as mean ± standard deviation. The same letter in the same figure means no significant differences among values (*p* < 0.05) according to the LSD’s test. (**A**) sinigrin; (**B**) gluconapin; (**C**) progoitrin; (**D**) neoglucobrassicin; (**E**) 4-methoxyglucobrassicin; (**F**) glucobrassicin; (**G**) 4-hydroxyglucobrassicin; (**H**) total aliphatic glucosinolates; (**I**) total indolic glucosinolates; (**J**) total glucosinolates. D1: dark treatment for one day; D2: dark treatment for two days; D3: dark treatment for three days; D4: dark treatment for four days.

**Figure 2:**
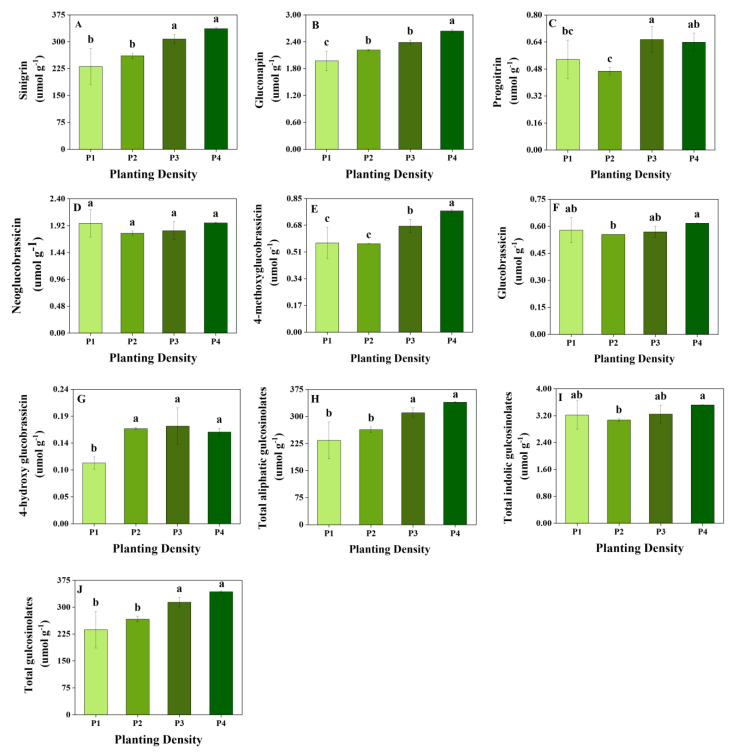
Glucosinolate content under different planting densities in mustard sprouts. Data are expressed as mean ± standard deviation. Same letter in the same figure means no significant differences among values (*p* < 0.05) according to the LSD’s test. (**A**) sinigrin; (**B**) gluconapin; (**C**) progoitrin; (**D**) neoglucobrassicin; (**E**) 4-methoxyglucobrassicin; (**F**) glucobrassicin; (**G**) 4-hydroxyglucobrassicin; (**H**) total aliphatic glucosinolates; (**I**) total indolic glucosinolates; (**J**) total glucosinolates. P1: a sowing density of 4 g of seeds per tray; P2: a sowing density of 5 g of seeds per tray; P3: a sowing density of 6 g of seeds per tray; P4: a sowing density of 7 g of seeds per tray.

Three aliphatic and four indolic glucosinolates were identified in mustard sprouts in our study. The content of total and individual glucosinolates varied among the dark day and planting density treatments (Figure 1 and Figure 2).

There was little variation in the content of total glucosinolates among the dark day treatments, especially aliphatic glucosinolates (Figure 1). No significant differences were observed in the content of sinigrin, which accounted for the greatest proportion of total glucosinolates; the same was the case for gluconapin and total aliphatic glucosinolates. The content of progoitrin was slightly higher in D2 (0.68 μmol g^−1^) compared with the other treatments. However, the highest levels of the indolic glucosinolates 4-hydroxy glucobrassicin (0.12 μmol g^−1^), glucobrassicin (0.76 μmol g^−1^), and neoglucobrassicin (2.46 μmol g^−1^) were all observed in D2; the only exception was the content of 4-methoxyglucobrassicin, which was highest in D4 (0.53 μmol g^−1^). The content of total indolic glucosinolates was 27.42%, 7.34%, and 9.39% higher in D2 (3.88 μmol g^−1^) than in D1 (3.05 μmol g^−1^), D3 (3.61 μmol g^−1^), and D4 (3.55 μmol g^−1^), respectively.

The content of aliphatic glucosinolates varied significantly among the planting density treatments (Figure 2). The highest level of sinigrin was observed in P4 (336.42 μmol g^−1^), followed by P3 (307.58 μmol g^−1^); the content of sinigrin in P4 and P3 was 45.41% and 32.94% higher than that in P1 and 28.93% and 17.88% higher than that in P2, respectively. The highest levels of gluconapin and progoitrin were observed in P4 (2.64 μmol g^−1^) and P3 (0.65 μmol g^−1^), respectively. The content of total aliphatic glucosinolates was higher in P4 (339.70 μmol g^−1^) and P3 (310.62 μmol g^−1^) than in P1 (233.87 μmol g^−1^) and P2 (263.62 μmol g^−1^); the same was the case for total glucosinolates. The variation in indolic glucosinolates was low among the planting density treatments. No significant differences were observed in the content of 4-hydroxy glucobrassicin and neoglucobrassicin among the planting density treatments, and the highest content of glucobrassicin (0.62 μmol g^−1^), 4-methoxyglucobrassicin (0.77 μmol g^−1^), and total indolic glucosinolate (3.52 μmol g^−1^) was observed in P4.

### 2.7. PCA

To evaluate the effect of treatment more comprehensively and systematically, PCA was performed to characterize differences in the content of the main health-promoting phytochemicals and antioxidant capacity among dark day and planting density treatments.

**Figure 3:**
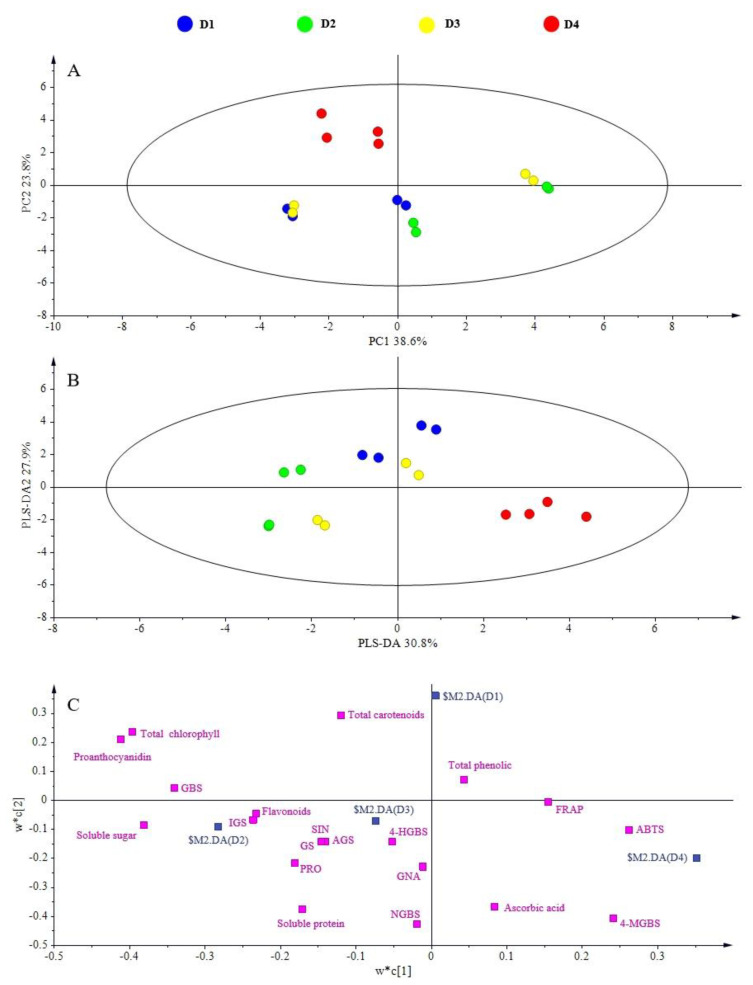
PCA analysis of different dark treatments on mustard sprouts. (**A**) PCA score plot; (**B**) PLS-DA score plot; (**C**) loading plot. SIN: sinigrin; GNA: gluconapin; PRO: progoitrin; NGBS: neoglucobrassicin; GBS: glucobrassicin; 4-MGBS: 4-methoxyglucobrassicin; 4-HGBS: 4-hydroxyglucobrassicin; AGS: total aliphatic glucosinolates; IGS: total indolic glucosinolates; GS: total glucosinolates. D1: dark treatment for one day; D2: dark treatment for two days; D3: dark treatment for three days; D4: dark treatment for four days.

The first principal component (PC1) and second principal component (PC2) explained 38.6% and 23.8% of the variance, respectively, for the dark day PCA. D2 and D4 were separated along PC1, and D4 was separated from D1 and D2 along PC2 (Figure 3A). PLS-DA was also performed. PLS-DA1 and PLS-DA2 accounted for 30.8% and 27.9% of the variance, respectively, and their distributions were similar to PC1 and PC2 (Figure 3B). According to the loading and VIP plots of the PLS-DA, the major contributors to D2 were soluble sugar, flavonoids, total indolic glucosinolates, glucobrassicin, total chlorophyll, and proanthocyanidins, and the major contributors to D4 were ABTS and 4-methoxyglucobrassicin (Figure 3C).

**Figure 4:**
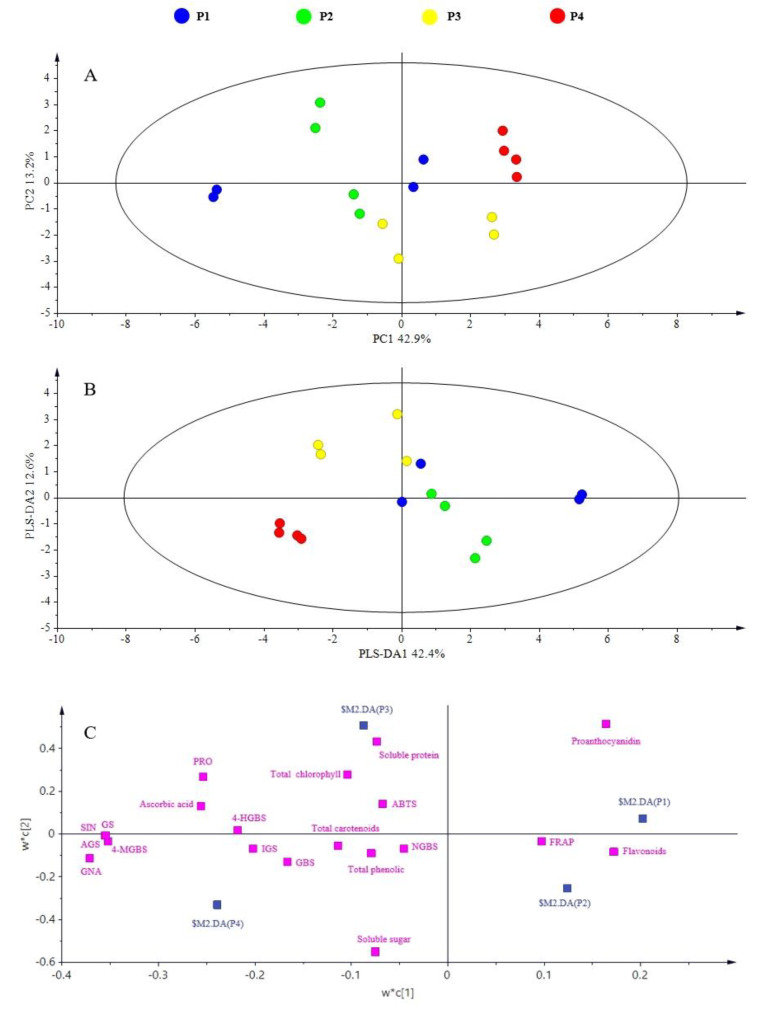
PCA analysis of different planting densities on mustard sprouts. (**A**) PCA score plot; (**B**) PLS-DA score plot; (**C**) loading plot. SIN: sinigrin; GNA: gluconapin; PRO: progoitrin; NGBS: neoglucobrassicin; GBS: glucobrassicin; 4-MGBS: 4-methoxyglucobrassicin; 4-HGBS: 4-hydroxyglucobrassicin; AGS: total aliphatic glucosinolates; IGS: total indolic glucosinolates; GS: total glucosinolates. P1: a sowing density of 4 g of seeds per tray; P2: a sowing density of 5 g of seeds per tray; P3: a sowing density of 6 g of seeds per tray; P4: a sowing density of 7 g of seeds per tray.

PC1 and PC2 explained 42.9% and 13.2% of the variance, respectively, in the planting density PCA. P4 and P2 were separated along PC1, and P4 was separated from P3 along PC2 (Figure 4A). PLS-DA was also performed. PLS-DA1 and PLS-DA2 accounted for 42.4% and 12.6% of the variance, respectively. P4 and P3 could be discriminated from P1 and P2 along PLS-DA1, and P4 could be discriminated from P3 along PLS-DA2 (Figure 4B). According to the loading and VIP plots of the PLS-DA, ascorbic acid, progoitrin, gluconapin, 4-methoxyglucobrassicin, sinigrin, total glucosinolates, and total aliphatic glucosinolates were the major contributors to P4, soluble protein was the main contributor to P3, and proanthocyanidins were the main contributor to P1 (Figure 4C).

### 2.8. Correlation Analysis

To investigate the correlations between the health-promoting phytochemicals and antioxidant activity, Pearson correlation coefficients were determined, and the correlation threshold R^2^ > 0.65 was used. Three groups with significant positive correlations among variables were detected. The first group included correlations among sinigrin, progoitrin, gluconapin, total aliphatic glucosinolates, and total glucosinolates; the second group included correlations among total phenolics, ascorbic acid, proanthocyanidins, FRAP, and 4-methoxyglucobrassicin; the last group included correlations among total indolic glucosinolates and glucobrassicin. No correlations were observed among the above three groups.

**Figure 5:**
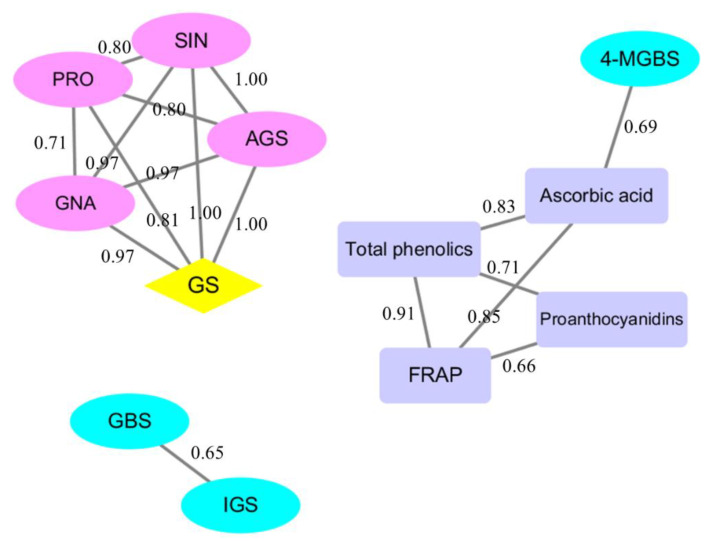
Correlation plot of the correlations between health-promoting phytochemicals and antioxidant capacity in mustard sprouts. All correlations in the figure reflect the absolute values of Pearson correlation coefficient above the threshold (R^2^ > 0.65). The numbers on the lines represent the specific value of the correlation coefficient. SIN: sinigrin; GNA: gluconapin; PRO: progoitrin; NGBS: neoglucobrassicin; GBS: glucobrassicin; 4-MGBS: 4-methoxyglucobrassicin; 4-HGBS: 4-hydroxyglucobrassicin; AGS: total aliphatic glucosinolates; IGS: total indolic glucosinolates; GS: total glucosinolates.

In the first group, total glucosinolates were positively correlated with total aliphatic glucosinolates, sinigrin, progoitrin, and gluconapin, and this group had the highest number of correlations (four edges). In the second group, FRAP and total phenolics were significantly correlated with each other and with ascorbic acid and proanthocyanidins (three edges), whereas the latter two were irrelevant. In addition, 4-methoxyglucobrassicin was correlated with ascorbic acid. In the third group, total indolic glucosinolates and glucobrassicin were positively correlated with each other (Figure 5).

## 3. Discussion

The health-promoting phytochemicals and antioxidant activity of mustard sprouts were greatly affected by the number of days they were exposed to darkness and planting density.

Dark conditions promote the skotomorphogenesis of sprouts. During this process, the hypocotyl rapidly elongates, the cotyledon slowly expands, and undifferentiated chloroplast precursors are produced [23]. This process is modulated by phytochrome-interacting factors (PIFs) to a large extent, suggesting that the length of exposure to dark treatment affects levels of photosynthetic pigments, such as chlorophyll and carotenoids [24]. Cotyledons are considered the main assimilatory organ in the early stage of sprouts, and their size, regulated by the length of dark exposure, is closely related to the accumulation of plant nutrients, such as soluble sugar and protein [23,25]. In the dark day experiment, D2 was optimal for maximizing the concentrations of chlorophyll, carotenoids, soluble sugar, and soluble protein in mustard sprouts. The content of chlorophyll and carotenoids did not vary significantly among planting density treatments; this might stem from the shading of tender leaves by neighboring plants in the early sprouting stage, which prevents light from being detected by the phytochrome photoreceptors [24,26]. Research has shown that planting density affects the content of soluble sugar and protein [27]. However, no linear relationship between planting density and the content of soluble sugar and protein in mustard sprouts was observed in our study.

The content of ascorbic acid and phenolic compounds (including total phenolics, flavonoids, and proanthocyanidins) and antioxidant activities were measured to characterize variation in antioxidant capacity among treatments [28]. In previous studies, radish sprouts grown under dark conditions were shown to have fewer total phenolic and flavonoid compounds than those grown under light conditions [29]. Phenolic compounds and antioxidant activities in broccoli and kale sprouts are lowest under constant darkness and increase with the duration of artificial light exposure [30]. The expression of the genes involved in polyphenol biosynthesis is highly upregulated by light stimulation when reactive oxygen species (ROS) accumulate under light stress [30,31]. This effect has also been observed in flavonoid compounds and other antioxidants [32]. However, in our study, the antioxidant capacity did not continuously decrease with the number of dark days. The content of ascorbic acid (1.41 mg g^−1^), ABTS^+^ (47.97%), and FRAP (0.15 mg g^−1^) was highest in D4; however, D2 was the best overall treatment compared with D1. Therefore, we speculated that appropriate levels of dark exposure are needed to optimize antioxidant capacity, and this requires further study [31]. Planting density has been shown to affect the antioxidant capacity, including the content of ascorbic acid and total phenolics, in previous studies [20,33]. The same has been observed for glucosinolates; abiotic stress caused by planting density is the main factor affecting the antioxidant capacity of plants [17,22,33]. Competition for nutrients and space increases with planting density, and this is thought to increase the antioxidant capacity [33,34]. However, in the planting density experiment, ascorbic acid and proanthocyanidins were the only compounds that were most abundant at high planting densities: P3 (6.39 mg g^−1^) and P4 (2.61 mg g^−1^), respectively. No significant variation in the other compounds was observed among planting density treatments, and this might suggest that the effect of planting density is negligible in the early sprouting stage of mustard.

Glucosinolates are important secondary metabolites in cruciferous plants that have received much interest in recent years for their strong biological activity and anti-cancer function [1,35]. Seven glucosinolates of two classes, aliphatic and indolic, were detected in mustard sprouts in this study, with sinigrin being predominant. The abundance of these glucosinolates might contribute to the high nutritional value of mustard sprouts [2,3]. However, the content of glucosinolates is heavily affected by environmental conditions and cultivation methods; it thus responded strongly to these two factors examined in our study [35].

There has been much debate regarding whether glucosinolate biosynthesis is stimulated by light or dark treatment [7]. Sprouts grown in the dark have a higher content of indolic glucosinolates, whereas those grown under light have a higher content of aliphatic glucosinolates [36,37]. Dark treatment had the strongest positive effect on total indolic glucosinolates compared with aliphatic glucosinolates. Glucobrassicin, an important indolic glucosinolate, is most abundant in 7-day-old mustard sprouts under complete darkness [36]. Rapeseed sprouts have higher concentrations of 4-hydroxyglucobrassicin, neoglucobrassicin, and total indolic glucosinolates when they are grown in the dark than when they are grown under light [36,38]. However, light treatment enhanced the concentrations of glucosinolates (expressed as mg of sinigrin per 100 g) by 33% in broccoli sprouts over 7 days of development [39]. The extent to which glucosinalbin (the specific and decisive aliphatic glucosinolate in white mustard) was reduced during germination was mitigated by treatment with 24 h of darkness [7]. The close correlation of glucosinolates with dark/light treatment might be related to the *R2R3-MYB* gene subfamily, a complex group of transcription factors in *Brassica* plants. The expression of *MYB34*, *MYB51*, and *MYB122*, which have a major effect on indolic glucosinolate biosynthesis, is upregulated by dark conditions, whereas the expression of *MYB28* and *MYB29*, which affect aliphatic glucosinolate biosynthesis, is upregulated under light exposure [37,40,41]. The expression levels of these transcription factors are also affected by the degree of light and dark regulation, depending on the intensity and length of darkness or light exposure [32,37,42]. Thus, for mustard sprouts, exposure to darkness for 2 d may maximize the upregulation of the expression levels of the transcription factors involved in glucosinolate regulation, especially indolic glucosinolates.

In the planting density experiment, the content of aliphatic and total glucosinolates increased with planting density. The concentrations of total aliphatic glucosinolates in P3 and P4 were 45.25% and 32.82% higher than that in P1 and 28.86% and 17.83% higher than that in P2, respectively; total glucosinolates were also significantly higher at high planting densities. The degradation of glucosinolate by myrosinase hydrolysis has been shown to be related to the response of plants to stress, and aliphatic glucosinolates are the most strongly affected by stress [43,44,45]. In the sprouts of polyploid *Brassica juncea*, the abundance of aliphatic glucosinolates is highest under biotic stress, such as glucose stress, and this is not the case with indolic glucosinolates [44]. An increase in glucosinolates, which is mainly determined by sinigrin, has been observed in broccoli in response to salt stress [45]. Additionally, as stress factors, nutrient and space deficiencies associated with high planting density are closely related to the content of glucosinolates. In six canola varieties, increases in plant densities result in a higher glucosinolate content [34]. Watercress grown under 20 cm spacing (72 plants per bed) is superior to 31 cm (36 plants per bed) spacing for maximizing the glucosinolate content [46]. Myrosinase-mediated glucosinolate degradation products can be rearranged to form different glucosinolate activation products, and this process is affected by planting density [43,47]. As planting densities increase, either direct elicitors from neighboring plants or indirect elicitors by competing nutrients and space lead to variation in the structural outcome of glucosinolate activation [43]. Thus, owing to nutrient and space stress, higher planting density (seeds at a density of 6–7 g) enhances yield as well as the nutritional value of mustard sprouts, and these changes are mostly associated with variations in glucosinolate concentrations [3,18].

## 4. Materials and Methods

### 4.1. Plant Material

The leaf mustard variant *Brassica juncea* var. *rugosa*, which is mostly cultivated for use as a fresh vegetable, was used as experimental material.

In the dark day experiment, 5 g of mustard seeds was evenly propagated in a seedling tray (32.6 cm × 22.4 cm × 4.1 cm) and covered with moist germinating paper. The mustard sprouts were exposed to darkness for 1 d (D1), 2 d (D2), 3 d (D3), and 4 d (D4) at a temperature of 25 °C and relative humidity (RH) of 70%. There were four replicates for each treatment, and one tray corresponded to one replicate. In the planting density experiment, 4 g (P1), 5 g (P2), 6 g (P3), and 7 g (P4) of mustard seeds were sown in each tray, and the plants were exposed to darkness for 2 d; all other conditions were the same among the treatments. After 7 d of growth, which corresponded to when the mustard sprouts reached the size at which they are typically harvested, the aerial parts were harvested, lyophilized, and stored at −20 °C for further analysis.

### 4.2. Soluble Protein Content

Fifty milligrams of freeze-dried powdered material was soaked in distilled water, and the solution was stirred for 30 s, settled for 30 min, and centrifuged for 5 min at 4000× *g*, and then 1 mL was transferred to a polypropylene tube. Coomassie brilliant blue G-250 was combined with 1 mL supernatant, and the absorbance was measured at 595 nm within 20 min after the reaction [4].

### 4.3. Soluble Sugar Content

Fifty milligrams of powder was extracted in 10 mL of distilled water for 20 min at 90 °C, and the homogenates were centrifuged at 4000× *g* for 5 min. A combination of 1 mL of sample extract, 0.5 mL of anthrone-ethyl acetate reagent, and 5 mL of concentrated sulfuric acid was homogenized and boiled for 5 min. The absorbance of the reaction mixtures was measured at 630 nm [4].

### 4.4. Chlorophyll and Carotenoids Content

An appropriate amount of sample was weighed, ground, and extracted with 10 mL of ethanol. The supernatant was collected and analyzed by the spectrophotometer, absorbance was detected at 665 nm, 649 nm, and 451 nm to measure the content of total chlorophyll and carotenoids, respectively [4].

### 4.5. Ascorbic Acid Content

Fifty mg of sample powder was extracted with 5 mL of 1.0% (*w*/*v*) oxalic acid and then centrifuged for 5 min at 4000× *g*. Each sample was filtered through a 0.45 μm cellulose acetate filter and analyzed by high-performance liquid chromatography (HPLC). The amount of ascorbic acid was calculated from absorbance values at 243 nm [4].

### 4.6. Proanthocyanidin Content

The powder of each sample was weighed, ground, and transferred to 4 mL of the extracting reagent. The solution was vortexed for 5 min, shaken for 1 h, and centrifuged at 4000× *g* for 5 min. The p-dimethylaminocinnamaldehyde reagent was added to 700 μL of supernatant. The absorbance of the mixture was spectrophotometrically detected at 640 nm after 20 min [48].

### 4.7. Flavonoid Content

Forty milligrams of sample powder was extracted in 50% ethanol and incubated at room temperature for 24 h in the dark. A 1.2 mL aliquot of the supernatant was mixed with 60 μL of 2% aluminum trichloride, 60 μL of 1 mol L^−1^ potassium acetate, and 1.68 mL of distilled water after being centrifuged. The absorption was read at 415 nm after 40 min [4].

### 4.8. Total Phenolic Content

Total phenolics were extracted with 50% ethanol, and the supernatant (300 μL) was mixed with 1.5 mL of 0.2 mol L^−1^ Folin–Ciocalteu reagent, and after 3 min, 1.2 mL of saturated sodium carbonate was added. The mixtures were allowed to stand for 20 min at room temperature, and the absorbance was measured at 760 nm with the spectrophotometer [4].

### 4.9. ABTS Assay

An aliquot of 300 μL of each extracted sample was added to 3 mL of ABTS^+^ solution. The absorbance was measured spectrophotometrically at 734 nm after exactly 2 h, and then the value was calculated [4].

### 4.10. Ferric Reducing Antioxidant Power (FRAP)

The extracted samples (300 μL) were added to 2.7 mL of the FRAP working solution, incubated at 37 °C, and vortexed. The absorbance was then recorded at 593 nm after 10 min, and the result was expressed as mmol kg^−1^ of dry weight [4].

### 4.11. Glucosinolate Composition and Content

Powdered samples (100 mg) were boiled in 5 mL of water for 10 min, and the supernatant was collected and applied to a DEAE-Sephadex A-25 column. The glucosinolates were converted into their desulpho analogues by treatment with aryl sulphatase, and the desulphoglucosinolates were eluted. HPLC analysis of desulphoglucosinolates was carried out using an Agilent 1260 HPLC instrument equipped with a variable wavelength detector (VWD) detector. The samples were separated at 30 °C on a Waters Spherisorb C18 column (250 × 4.6 mm) using acetonitrile and water at a flow rate of 1.0 m Lmin^−1^. The absorbance was detected at 226 nm [4].

### 4.12. Statistical Analyses

All assays were performed in four replicates and all results were shown as mean ± standard deviation. Statistical analysis used SPSS version 20 (SPSS Inc., Chicago, IL, USA). The data obtained were subjected to analysis of one-way ANOVAs. The histogram graphs were conducted using Origin 8.5.0 (OriginLab Corporation, Northampton, MA, USA). A principal components analysis (PCA) and partial least-squares discriminant analysis (PLS-DA) were performed using SIMCA 14.1 Demo software (Umetrics, Malmö, Sweden) with unit variance scaling to determine the relationships among the samples. The correlation analyses were also performed and were visualized using Cytoscape v. 3.5.1 (The Cytoscape Consortium, New York, NY, USA) [49,50].

## 5. Conclusions

Dark treatment and planting density had marked effects on the health-promoting phytochemicals and antioxidant capacity of mustard sprouts in our study. D2 increased the content of soluble sugar and protein, chlorophyll, carotenoids, and indolic glucosinolates. High planting densities (i.e., seeds sown at a density of 6–7 g per tray (P3 and P4) significantly enhanced the content of soluble sugar, protein, and aliphatic and total glucosinolates, and no marked increases were observed in the content of chlorophyll and carotenoids and antioxidant capacity. Overall, a sowing density of 6 g of seeds per tray and dark treatment for two days are optimal for maximizing the nutritional quality of mustard sprouts and minimizing costs. In addition, the regulatory mechanisms of darkness and planting density on indolic and aliphatic glucosinolates, respectively, in mustard sprouts, should be studied further. It is also of great significance to establish a quantifiable formula to more systematically evaluate the treatment effect in the future.

## Figures and Tables

**Table 1:** Soluble sugar, soluble protein, chlorophyll, and carotenoids content in different days of dark treatment of mustard sprouts (mg g^−1^).

Dark Days	Soluble Sugar	Soluble Protein	Total Chlorophyll	Total Carotenoids
D1	142.68 ± 5.06 ^c^	98.47 ± 1.69 ^c^	6.89 ± 0.13 ^b^	0.64 ± 0.02 ^a^
D2	158.36 ± 2.13 ^a^	109.14 ± 3.98 ^ab^	7.29 ± 0.06 ^a^	0.63 ± 0.01 ^ab^
D3	149.60 ± 6.29 ^b^	109.68 ± 5.64 ^a^	6.44 ± 0.24 ^c^	0.61 ± 0.04 ^ab^
D4	136.87 ± 2.28 ^c^	103.86 ± 0.76 ^bc^	5.70 ± 0.14 ^d^	0.60 ± 0.03 ^b^

Data are expressed as mean ± standard deviation. Same letter in the same column means no significant differences among values (*p* < 0.05) according to the LSD’s test. D1: dark treatment for one day; D2: dark treatment for two days; D3: dark treatment for three days; D4: dark treatment for four days.

**Table 2:** Soluble sugar, soluble protein, chlorophyll, and carotenoids content in different planting densities of mustard sprouts (mg g^−1^).

Planting Density	Soluble Sugar	Soluble Protein	Total Chlorophyll	Total Carotenoids
P1	138.74 ± 5.23 ^bc^	108.48 ± 4.15 ^ab^	6.38 ± 0.13 ^a^	0.63 ± 0.02 ^a^
P2	149.49 ± 8.74 ^ab^	107.35 ± 1.08 ^b^	6.30 ± 0.20 ^a^	0.67 ± 0.09 ^a^
P3	131.53 ± 9.44 ^c^	113.35 ± 4.44 ^a^	6.48 ± 0.08 ^a^	0.67 ± 0.04 ^a^
P4	152.65 ± 6.73 ^a^	108.09 ± 2.66 ^b^	6.40 ± 0.08 ^a^	0.68 ± 0.06 ^a^

Data are expressed as mean ± standard deviation. Same letter in the same column means no significant differences among values (*p* < 0.05) according to the LSD’s test. P1: a sowing density of 4 g of seeds per tray; P2: a sowing density of 5 g of seeds per tray; P3: a sowing density of 6 g of seeds per tray; P4: a sowing density of 7 g of seeds per tray.

**Table 3:** Ascorbic acid, proanthocyanidins, flavonoids, total phenolics, and antioxidant activity content in different days of dark treatment of mustard sprouts.

Dark Days	Ascorbic Acid (mg g^−1^)	Proanthocyanidins (mg g^−1^)	Flavonoids (mg g^−1^)	Total Phenolics (mg g^−1^)	ABTS^+^ (%)	FRAP (mmol g^−1^)
D1	1.02 ± 0.14 ^b^	5.71 ± 0.12 ^b^	13.73 ± 0.48 ^ab^	16.00 ± 0.58 ^a^	41.26 ± 3.05 ^ab^	0.14 ± 0.00 ^ab^
D2	1.31 ± 0.26 ^a^	5.95 ± 0.14 ^a^	14.92 ± 0.80 ^a^	16.05 ± 0.35 ^a^	39.86 ± 4.20 ^b^	0.14 ± 0.01 ^ab^
D3	1.24 ± 0.20 ^ab^	5.59 ± 0.04 ^b^	13.75 ± 1.43 ^ab^	14.96 ± 0.30 ^b^	38.55 ± 1.97 ^b^	0.14 ± 0.00 ^b^
D4	1.41 ± 0.14 ^a^	4.92 ± 0.12 ^c^	13.38 ± 0.44 ^b^	15.98 ± 0.83 ^a^	47.97 ± 7.51 ^a^	0.15 ± 0.01 ^a^

Data are expressed as mean ± standard deviation. Same letter in the same column means no significant differences among values (*p* < 0.05) according to the LSD’s test. D1: dark treatment for one day; D2: dark treatment for two days; D3: dark treatment for three days; D4: dark treatment for four days.

**Table 4:** Ascorbic acid, proanthocyanidins, flavonoids, total phenolics, and antioxidant activity content in different planting densities of mustard sprouts.

Planting Density	Ascorbic Acid (mg g^−1^)	Proanthocyanidins (mg g^−1^)	Flavonoids (mg g^−1^)	Total Phenolics (mg g^−1^)	ABTS^+^ (%)	FRAP (mmol g^−1^)
P1	2.24 ± 0.21 ^ab^	6.32 ± 0.15 ^ab^	15.02 ± 0.47 ^a^	18.27 ± 0.47 ^a^	39.07 ± 3.44 ^a^	0.17 ± 0.00 ^a^
P2	2.07 ± 0.24 ^b^	6.20 ± 0.09 ^bc^	15.57 ± 1.64 ^a^	18.96 ± 0.40 ^a^	41.30 ± 1.77 ^a^	0.17 ± 0.00 ^a^
P3	2.52 ± 0.25 ^a^	6.39 ± 0.09 ^a^	14.60 ± 0.51 ^a^	18.90 ± 0.84 ^a^	42.21 ± 1.55 ^a^	0.17 ± 0.00 ^a^
P4	2.61 ± 0.30 ^a^	6.04 ± 0.13 ^c^	14.32 ± 0.48 ^a^	19.13 ± 0.94 ^a^	40.30 ± 3.18 ^a^	0.17 ± 0.00 ^a^

Data are expressed as mean ± standard deviation. Same letter in the same column means no significant differences among values (*p* < 0.05) according to the LSD’s test. P1: a sowing density of 4 g of seeds per tray; P2: a sowing density of 5 g of seeds per tray; P3: a sowing density of 6 g of seeds per tray; P4: a sowing density of 7 g of seeds per tray.

## Data Availability

The data presented in this study are available in the manuscript and Supplementary Materials.

## Supplementary Materials

The following supporting information can be downloaded at: https://www.mdpi.com/article/10.3390/plants11192515/s1, Table S1: Correlation of the health-promoting phytochemicals and antioxidant capacity in mustard sprouts.
